# Potassium Iodide-Modified Lead-Free Cs_3_Bi_2_I_9_ Perovskites for Enhanced High-Efficiency Solar Cells

**DOI:** 10.3390/nano12213751

**Published:** 2022-10-25

**Authors:** Shindume Lomboleni Hamukwaya, Huiying Hao, Melvin Mununuri Mashingaidze, Tingting Zhong, Shu Tang, Jingjing Dong, Jie Xing, Hao Liu

**Affiliations:** 1 School of Science, China University of Geosciences, Beijing 100083, China; 2 Department of Mechanical & Metallurgical Engineering, School of Engineering & the Built Environment, University of Namibia, Ongwediva 33004, Namibia

**Keywords:** Cs_3_Bi_2_I_9_ perovskite solar cells, KI incorporation, crystallinity, morphology, cell stability

## Abstract

Lead-free, bismuth-based perovskite solar cells (PSCs) are promising, non-toxic, and stable alternatives to lead-based PSCs, which are environmentally harmful and highly unstable under deprived air conditions. However, bismuth-based PSCs still suffer from low-power-conversion efficiency (PCE) due to their large bandgap and poor film morphology. Their poor film-forming ability is the greatest obstacle to Cs₃Bi₂I₉ progress in thin-film solar cell technology. This study synthesizes novel, lead-free perovskites with a small bandgap, excellent stability, and highly improved photovoltaic performance by integrating different amounts of potassium iodide (KI) into a perovskite precursor solution. KI incorporation improves the crystallinity of the perovskite, increases the grain size, and decreases the potential contact distribution, which is demonstrated by X-ray diffraction, electronic scanning microscopy, atomic force microscopy, and ultraviolet-visible spectroscopy. The Cs₃Bi₂I₉ PSC device with 2 vol. % incorporation of KI shows the highest PCE of 2.81% and Voc of 1.01 V as far as all the Bi-based cells fabricated for this study are concerned. The study demonstrates that incorporating KI in the Cs₃Bi₂I₉ perovskite layer highly stabilizes the resultant PSC device against humidity to the extent that it maintains 98% of the initial PCE after 90 days, which is suitable for solar cell applications. The devices also demonstrate greater resistance to airborne contaminants and high temperatures without encapsulation, opening up new possibilities for lead-free Cs₃Bi₂I₉ PSCs in future commercialization.

## 1. Introduction

Excellent optoelectronic characteristics (such as bandgap tuning, interfacial carrier mobility, and extremely lengthy diffusion times for charge carriers are only a few of the features discussed in the literature), low cost, and ease of preparation at low temperatures make metal halide perovskites prospective competitors for leading photovoltaic application technologies [1,2,3,4]. PSCs, such as lead-based iodide PSCs, are promising technologies that have already achieved more than 25.5% power conversion efficiency (PCE) in recent years [5,6,7,8], making them comparable to commercialized thin-film counterparts [9]. Such photovoltaic cells have short lives, attributed to chemical instability. The existence of toxic Pb in perovskites with a general chemical formula of APbX_3_ limits potential commercialization, especially considering their poor stability [10,11]. Therefore, Pb-based perovskite materials for optoelectronic applications are constrained and required to replace Pb with a non-toxic element [1,12].

Limiting ion transportation, passivating surfaces, adjusting compositions, and synthesizing low-dimensional perovskite materials are only some of the techniques employed to enhance long-term stability [12,13,14]. Substituting Pb^2+^ with trivalent cations like Sn^2+^ and Ge^2+^ is the only proven method of eliminating Pb toxicity. However, the crystal lattices of Sn^2+^ or Ge^2+^-based perovskites are readily destroyed by the oxidation of quadrivalent molecules in the air atmosphere, making these perovskites highly unstable [15]. Two Pb^2+^ sites are being replaced by Sn^4+^, Ge^4+^, Pd^4+^, or Ti^4+^, while three Pb^2+^ sites are being replaced by two metal cations, for example, Bi^3+^ or Sb^3+^ [1,16]. This appears to be how the organized metallic vacancies keep their charge neutrality while having smaller electronic dimensions and slower carrier movement [17]. To strike a middle ground between cell efficiency and Pb toxicity, developing novel three-dimensional double perovskite analogs appears essential.

Since the structural properties of perovskite are determined by the 6s^2^6p^0^ configuration of Pb^2+^, the accessible replacement element is Bi^3+^ due to its comparable electronic distribution and lone-pair electron compared to Pb^2+^ [18,19]. A₃Bi_2 × 9_ is the general structural formula for Bi-based perovskites, where A represents cations, such as Na^+^, K^+^, Rb^+^, Cs+, or CH₃NH^3+^ (methyl ammonium (MA)), and X represents a halogen element (F, Cl, Br, or I) [20]. The Bi^3+^ cation is a low-temperature processable solution, has a significant dielectric constant, and has a stable 6p-block structure, all of which make it non-toxic. A reduced, inherent trap density and defect state in the Bi-based perovskite allows for a prolonged photogenerated charge lifetime [21]. Bi-based halides, another defect-tolerant material, are likewise predicted to have an octahedral-coordinated structure [22]. Since Bi-based perovskites are more stable in humid settings than Pb-based halide perovskites, they have attracted much consideration as a potential substitute for Pb-based perovskites [12,23].

Much interest has been paid to all-inorganic bismuth-based perovskites because of their superior stability, low environmental impact, and inexpensive solution processability. However, the efficiency of a Bi-based perovskite photodetector lags significantly behind Pb-based perovskites due to the substantial electron-hole pair-binding energy and the limited light-absorption coefficient. Most of the earlier research on Bi-based perovskite materials centered on crystal structure and phase transition [24], and therefore, there are few publications on their application as perovskite light-absorber materials in photovoltaic cells. Park et al. synthesized the Bi-based perovskite A_3_Bi_2_I_9_ (A designating Cs and methylammonium (MA)) as a photovoltaic absorber, ushering in a new era in the field of perovskites [25]. The best PCE for PSCs recorded from a Bi base is 3.20% [26]. There are several possible causes for the low efficiency, including additional bandgap states, suboptimal morphology, an overabundance of reactant residue, or a lack of interfacial contact.

Interestingly, the perovskite-structure A-site organic ion is sometimes partially replaced by alkali metals with tiny ionic radii, such as K, Rb, and Cs [27,28]. Alkali metals are often added to the perovskite’s structure to reduce hysteresis and increase the PSC’s stability. Undoubtedly, adding potassium iodide (KI) will boost the efficiency of PSCs. According to Tang et al., KI inclusion can alter the perovskite’s band orientation, leading to a lower charge-transfer barrier. As a result, the hysteresis effect disappears [29], and Bu et al. reported that KI reduced hysteresis and improved conductivity [30]. With the inclusion of rubidium (Rb⁺), Saliba et al. investigated a triple-cation mixed-halide perovskite that demonstrated improved device performance in a solar device [31]. However, experiments on triple cations with Rb^⁺^ and MAPbI₃ showed that their performance improved when K⁺ was added, indicating that such additives are not absorbed into the perovskite lattice and instead experience segregation, generating secondary phases [32]. Bismuth is a heavy specialty metal with a density of 9.8 g/cc, mainly found in minor concentrations in the Earth’s crust (21,000 ppm) as a native metal, with the bulk of commercially traded bismuth a by-product of refining base- and precious-metal concentrates [29]. It is mainly used for alloying with other metals and in compound form for organic synthesis and cosmetic and therapeutic functions. Commercializing Bi-based PSCs would create a sustainable application and considerable demand for this environmentally benign and non-toxic element, which is often regarded as a nuisance in lead, copper, and zinc smelters that they charge penalties based on its concentration.

KI has been shown to be an excellent defect-passivating additive in triple-cation perovskites [22]. It hypothesized that the KI’s surplus iodide species would preserve the perovskite’s halide vacancies, creating materials with a high light-absorption quantum efficiency and significantly lower photo-induced halide separation. However, the photo-induced halide separation was attenuated by a potassium halide phase that was located in the grain boundary region. According to Abdi-Jalebi et al., perovskites are particularly additive-tolerant; adding new materials may lead to a better photovoltaic performance [33]. There is no need to add a second layer, such as in many other photovoltaic technologies to enhance the performance; instead, the additive is merely incorporated into the perovskite solution [33]. The ability of next-generation solar cells to convert more sunlight into electricity thanks to a simple potassium treatment could enhance their efficiency. The flaws and immobilized ion mobility, which have lately restricted the effectiveness of affordable PSCs, were rehabilitated by adding potassium iodide. These next-generation solar cells may be created as independent photovoltaic devices or colored LEDs, or they may be used to increase the efficiency of existing silicon-based solar cells.

Perovskite solar cell (PSC) luminescence efficiencies are still significantly less than 100%, so there is an opportunity for development. Photo-induced ion migration and parasitic non-radiative losses are the leading causes of low luminescence, which are reduced in perovskites with the potassium halide layer on their surfaces and grain boundaries [34]. KI rehabilitates the traps so the electrons can move more freely and immobilize the ions, making the material more stable at the appropriate bandgap. Potassium manages the ions and defects in perovskites, stabilizing the bandgaps and increasing luminescence, leading to more effective solar cells. Researchers used this method on various mixed-halide perovskites, producing luminescence close to the efficiency thresholds and enhancing the charge transfer and electrode interface.

In this work, the researchers change the chemical makeup of inorganic, lead-free Cs₃Bi₂I₉ perovskite layers by incorporating KI into the Cs₃Bi₂I₉ perovskite solution before fabricating the solar cells using a one-step spin-coating technique, such that the KI forms a surface layer on top of the perovskite. They study the effects of varying the concentration of KI from 0 v% to 10 v% in step increases of 2% on the fabricated PSCs’ structural, morphological, and optoelectronic characteristics, photovoltaic performance, and stability. The synthesized KI-incorporated Cs₃Bi₂I₉ perovskite layer is successfully utilized in FTO/c-TiO₂/M-TiO₂/Cs₃Bi₂I₉/C solar cells. A maximum PCE of 2.81%, Voc of 1.01V, Jsc of 3.60 mA.cm^−2^, and an FF of 77% are observed in the Cs₃Bi₂I₉ PSC with 2 vol% KI incorporation, which is significantly less than the highest previously reported values of 3.59% [17] and 3.20% [26], but good enough to warrant further studies on improving this PCE, especially for large-area bismuth PSCs.

## 2. Materials and Methods

### 2.1. Materials

All the PSCs fabrication processes were conducted in ambient conditions (RH: 20%–50%, room).

The laser-patterned FTO (TEC-A7) glass substrate and TiO₂ paste (Dyesol-30-NR-D) were purchased from Advanced Election Technology Co., Ltd. (Youxuan Tech, Liaoning, China). Titanium (IV) isopropoxide (99.999%), ethanol (≥99.5%), dimethyl sulfoxide (DMSO, 99.8%), bismuth (III) iodide (BiI₃ 98%), potassium iodide (KI 99.99%), and silver iodide (AgI 99%) were purchased from Aladdin Corporation (Shanghai, China). Cesium iodide (CsI) was obtained from Xi’an Polymer Light Technology Corp. Carbon paste was procured from Shanghai MaterWin New Materials Co., Ltd., Shanghai, China.

### 2.2. Preparation of Electron Transport Layer

This method was adopted from our previous work [14]; the fluorine-doped tin oxide (FTO) glass substrates were sequentially cleaned with acetone, isopropanol, anhydrous alcohol, and deionized water in a sequential ultrasonic cleaner for 15 min, followed by a 5 min plasma-cleaning treatment for 50 W.

A total of 80 μL of titanium(IV) isopropoxide and 16 μL of concentrated hydrochloric acid were mixed in 200 μL of ethanol and stirred for 30 min at room temperature to compound the compact TiO₂ (C-TiO₂).

Subsequently, the C-TiO₂ layers were deposited by the spin-coating method on the FTO glass substrates at 3000 rpm for 30 s using a two-step method and then annealed at 150 °C for 5 and 15 min, respectively, followed by substrate heated treatment at 500 °C for 30 min in a muffle furnace. After cooling, the substrates were placed in a 0.04 M titanium tetrachloride (TiCl_4_) aqueous solution and kept in the oven for 30 min at 70 °C. They were then rinsed with deionized water, dried, and annealed at 500 °C in a muffle furnace for 30 min. Then, the layer of compact titanium dioxide was successfully prepared.

The mesoporous titanium dioxide (M-TiO₂) precursor was prepared by mixing TiO₂ paste with ethanol (weight ratio = 1:4). The M-TiO₂ layer was spin-coated on the FTO substrate with a C-TiO₂ layer at a speed of 4000 rpm for 40 s, kept at 80 °C for 40 min in an oven, followed by heat treatment in a muffle furnace at 500 °C for 30 min. After cooling, the substrate was placed in a 0.02 M TiCl_4_ aqueous solution and kept at 70 °C for 30 min, followed by rinsing with deionized water, dried, and then annealed at 500 °C in a muffle furnace for 30 min. A layer of mesoporous titanium dioxide was thus successfully fabricated on the substrate.

### 2.3. Perovskite Solution Preparation and Device Fabrication

First, 2.48 M CsI, and 1.72 M BiI₃ salts were prepared by dissolving pre-determined amounts in DMSO solution. The solution was stirred for 24 h at 70 °C in an oil bath. A total of 2 M of KI was prepared as a stock solution, then different volume percentages (0 vol%, 2 vol%, 4 vol%, 6 vol%, 8 vol%, and 10 vol%) of the stock solution were added to the perovskite solution in appropriate volume ratios and stirred for 6 h.

**Figure 1:**
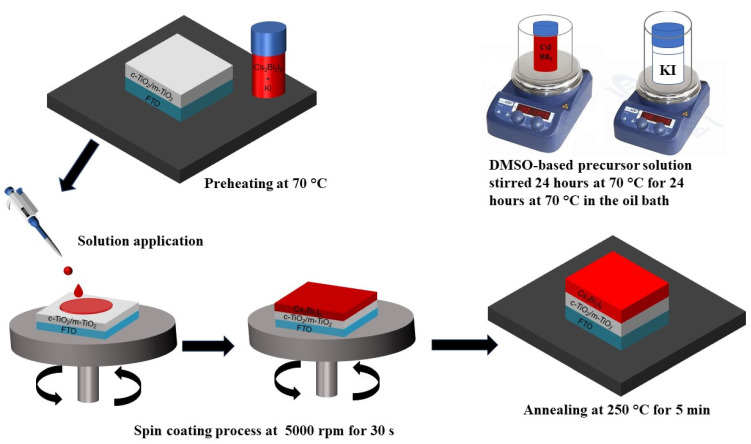
Schematic illustration of the conventional spin-coating process for preparing the Cs_3_Bi_2_I_9_ and Cs_3_Bi_2_I_9_ KI-incorporation devices.

The Cs₃Bi₂I₉ perovskite film was fabricated by a one-step spin-coating method of a mixture of Cs₃Bi₂I₉ and KI solutions on the FTO/c-TiO₂/M-TiO₂-layer substrate was preheated at 70 °C at 5000 rpm for the 30 s, and then constantly annealed at 250 °C for 5 min, as shown in Figure 1. After cooling, the ternary-cation Cs₃Bi₂I₉ PSCs incorporated KI-absorption layer was completely prepared. Finally, using the purchased carbon-electrode paste and mask, the carbon electrode was pasted onto the absorption layer, with each cell-electrode area fixed at 0.01 cm^2^ and then annealed on a hot plate at 120 °C for 15 min under ambient air conditions. Then, the PSC device was successfully prepared.

The prepared Cs₃Bi₂I₉ perovskite samples were labeled as K-0, K-2, K-4, K-6, K-8, and K-10 (where 0, 2, 4, 6, 8, and 10 correspond to 0 vol %, 2 vol %, 4 vol %, 6 vol %, 8 vol %, and 10 vol %, respectively of KI added). The K-0 PSC devices were used for the control experiments.

### 2.4. Film and Device Characterizations

Current-density−voltage (J−V) characteristics were studied by using a source meter (Keithley 2400, Keithley Instruments China, Beijing, China) under a simulated solar illumination of 100 mW cm^−2^ (AM 1.5G) by scanning at a rate of 100 mV/s for 1.4 to −0.2 V in the air (Zolix SCS10-X150-DZ Solar Simulator system, Zolix, Beijing, China). The devices were illuminated for 2 to 3 min before scanning. The surface and cross-sectional morphologies of the PSCs devices were performed by scanning electron microscopy (SEM, S-4800, Hitachi, Tokyo, Japan). A Bruker Dimension Edge Atomic Force Microscope (AFM) was used to characterize the perovskite film, while X-ray diffraction (XRD) patterns were obtained from the samples of perovskite films using an X-ray diffractometer with Cu K-α radiation (Bruker, Karlsruhe, Germany). UV−Vis absorption spectra of the perovskite films were tested on a spectrophotometer (Cary 5000, Palo Alto, CA, USA). The steady-state photoluminescence (PL) spectra were obtained using the PL spectrometer (Fluoro-Max-4, Horiba, Edison, NJ, USA) at an excitation wavelength of 510 nm.

## 3. Result and Discussion

**Figure 2:**
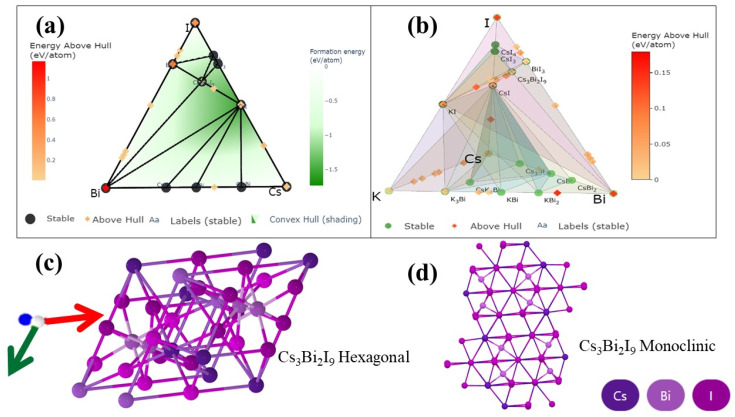
(**a**) Phase diagram of the Cs-Bi-I ternary system. (**b**) Phase diagram of the Cs-Bi-I-K quaternary system. (**c**) Crystal structures of the three-dimensional perovskite *P*6₃/mmc Cs₃Bi₂I₉. (**d**) Crystal structures of the three-dimensional perovskite C2/c Cs₃Bi₂I₉.

Figure 2 shows the thermodynamic phase transformation of the multi-component system’s phase diagram. It is a valuable tool in material engineering for determining the thermodynamic stability of compounds, projected equilibrium chemical reactions, and processing methods for material synthesis. Phase diagrams are useful tools for understanding chemical systems, but their experimental determination is time-consuming due to the need for the precise synthesis and characterization of all phases in a thermodynamic system [35].

Figure 2a,b show the three-dimensional structure of Cs₃Bi₂I₉ perovskite crystals. Figure 2b shows that the phase diagram of potassium iodide-modified Cs₃Bi₂I₉ PSCs have two crystal systems: hexagonal and monoclinic [36]. The numbers of atoms in the Cs₃Bi₂I₉ perovskites in the primitive cell hexagonal and monoclinic are 28, as shown in Table 1. The Bi atom is located at the center of the octahedron in the Cs₃Bi₂I₉ perovskites and is surrounded by six iodide anions, unlike lead-based perovskites, where the two octahedrons share three iodine atoms. In Figure 2c, Cs₃Bi₂I₉ crystallizes in the hexagonal P6₃/mmc space group. There are two inequivalent Cs⁺ sites. In the first Cs⁺ site, Cs⁺ is bonded to twelve I⁻ atoms to form CsI₁₂ cuboctahedra that share corners with nine CsI₁₂ cuboctahedra, corners with three equivalent BiI₆ octahedral faces with seven CsI₁₂ cuboctahedra, and faces with four equivalent BiI₆ octahedra. Figure 2d illustrates that Cs₃Bi₂I₉ crystallizes in the monoclinic C2/c space group. The structure is three-dimensional.

Table 1 shows two polymorphs in trivalent cation-based Cs₃Bi₂I₉ lead-free perovskites: 0-dimensional (dimer, P6_3_/mmc) and 2-dimensional (layered, P3m1) phases. The 2D phase is favored for carrier transport [1,37] due to its narrower bandgap, although maintaining this phase at an ambient temperature is difficult.

### 3.1. Device Composition Analysis

**Figure 3:**
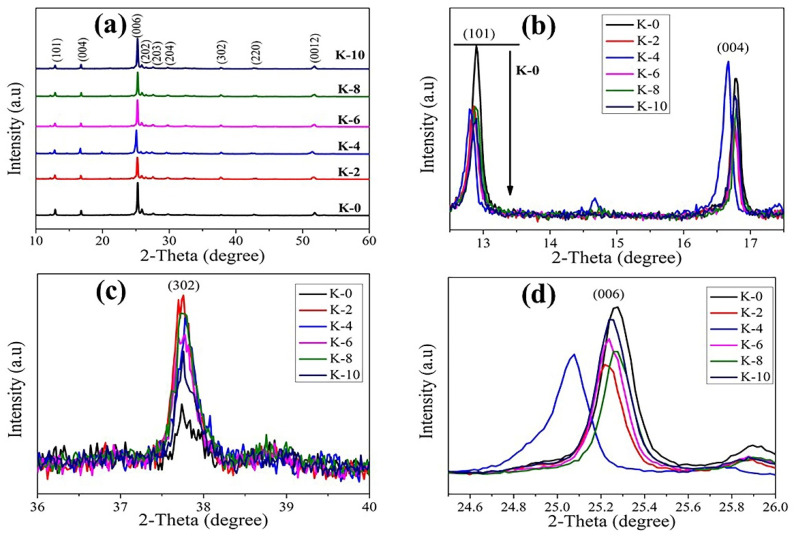
(**a**) XRD patterns of Cs₃Bi₂I₉ and Cs₃Bi₂I₉ perovskite devices with different concentrations of KI, (**b**) magnified view of XRD patterns of prepared perovskite devices (K = 0–10 vol%) show a decrease in BiI₃ peak intensity around 2θ = 12.7°, (**c**) magnified view of XRD patterns at 37.758° (302), and (**d**) variation in (006) peak around 2θ = 25.259° with KI incorporation.

X-ray diffraction (XRD) data were the first to confirm the existence of the Cs₃Bi₂I₉ crystal structure. Perovskite precursor solution of as-prepared Cs₃Bi₂I₉ was deposited on FTO/c-TiO₂/M-TiO₂ by a one-step spin-coating process, and their XRD patterns are displayed in Figure 3. According to Table 1, all of the Cs₃Bi₂I₉ perovskite devices are polycrystalline and exhibit a hexagonal structure with a spatial family of P6_3_/mmc [26].

Figure 3 displays the XRD patterns obtained when studying the Cs₃Bi₂I₉ PSCs samples with different concentrations of KI incorporated into the absorber films, and shows the effect of KI on the perovskite film-phase crystallization. For the PSC film without KI (K-0), the diffraction peaks observed at 2θ = 12.838 (101) and 25.208 (006) indicate the presence of excess unreacted BiI₃ [38]. The addition of KI systematically decreases the intensity of BiI₃ peaks, as can be observed in Figure 3a,b, which is in agreement with potassium passivation [39]. Intense peaks at 2θ = 12.838°, 16.713°, 25.208°, 25.802°, 27.506°, 29.96°, and 51.751° correspond to the (101), (004), (006), (202), (203), (204), and (0012) planes of the Cs₃Bi₂I₉ lattice and are characteristic of this perovskite phase.

The XRD patterns exhibited sharp diffraction peaks, indicating that the ternary Cs₃Bi₂I₉ perovskite structures generated were highly crystalline. The XRD data acquired were a good match with the historic JCPDS card number 23-0847 [40]. Despite the poor resolution, peaks that match these planes can be seen in the experimental data. The size of the nanocrystals may be to blame for the prominent peaks, but the many little peaks within ranges of 2θ observed in the reference pattern were also possible causes. It has been previously stated that broad signals obtained in nanocrystal production can be attributable to tiny particle sizes. Because of this, we know that the Pb-free perovskite structures were formed successfully.

As seen in Figure 3c, peak shift is typically more noticeable at greater angles. The 37.758° hkl (302) peak was carefully scanned to observe the extremely few changes in peak positions. The (302) peak of the K-0 sample shifts towards a lower angle, and this demonstrates that iodine occupies an interstitial location and causes a lower angle shift. The K-2 sample displays a more pronounced shift and has the maximum intensity compared to the other samples, indicating that the as-prepared perovskite film’s crystalline structure is significantly larger. It suggests that too much iodine causes halide exchange even when potassium is present. This explains why the K-2 sample exhibits greater lattice (hexagonal) expansion than the samples K-4, K-6, K-8, and K-10, which contain extra iodine. In summary, the lattice expansion in the Cs₃Bi₂I₉-containing perovskites of the K-2 sample is due to halide exchange and interfacial absorption of extra iodine.

**Figure 4:**
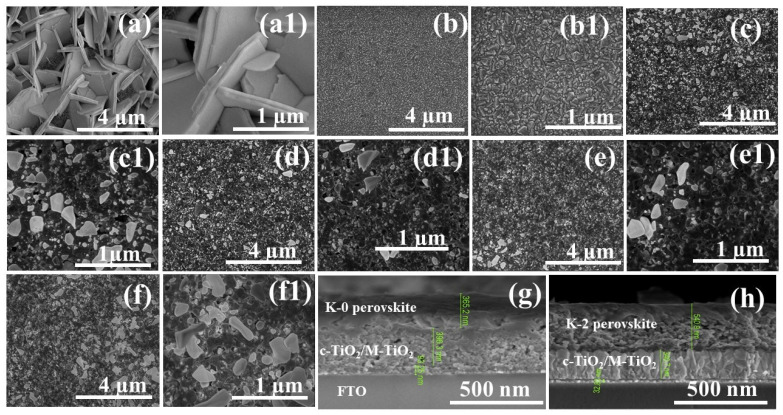
SEM images of Cs₃Bi₂I₉: (**a**) surface-top view morphologies of K-0 perovskite films, (**a1**) highly magnified view of (**a**), and (**b**–**f**) surface-top view morphologies of K-2 to K-10 perovskite films. (**b1**–**f1**) Highly magnified view of (**b**–**f**) K-2 to K-10. (**g**) Cross-sectional view of K-0 perovskite films. (**h**) Cross-sectional view of the best-performing device with K^+^-incorporated (K-2) perovskite films.

A magnified view of the scattering peak around 2θ = 25.259° in hexagonal Cs₃Bi₂I₉ perovskite (006) is shown in Figure 3d. Hence, with the measured addition amount of KI, the scattering strength of the (006) peak decreases, and the peak’s 2θ value is reduced. This suggests that the inclusion of KI increases the volume of the unit cell and decreases the measurement-axis of the coherent scattering domain size. On the other hand, it could indicate a decrease in grain size, which is consistent with the grain-size analysis based on SEM imaging [33], as depicted in Figure 4. The scattering intensity of X-rays decreased with the addition of KI for all peaks except for the K-10 sample, where scattering from the (006) planes was observed to be stronger. This suggests that the addition of KI changed nucleation and development, leading to improved orientation along the (006) plane. The observed growth in lattice size is associated with potassium being absent from incorporated cationic sites, as K^+^ has an ionic radius (1.38 Å) larger than Bi^3+^ (1.03 Å) and lesser ionic radius in comparison to the other cations in the ternary-cation composition (I^-^: 2.20, and Cs^+^ 1.67 Å) [41]. It was concluded that all the prepared Cs₃Bi₂I₉ perovskite films were extremely crystalline regardless of KI concentration [42], as evidenced by strong peaks in the XRD pattern, with a preferred orientation of 2θ = 25.208° along the (006) plane along the *x*-axis.

### 3.2. Morphological Structure

As depicted in Figure 4, scanning electron microscopy (SEM) was utilized to examine the surface topography and morphology of all prepared Cs₃Bi₂I₉ perovskite films that had just been deposited on FTO/c-TiO₂/M-TiO₂. In the SEM images taken at various resolutions, several microscopic and well-organized hexagonal crystals with a diameter varying from 4–1 µm were seen on the whole substrate surface of the Cs₃Bi₂I₉ perovskite film. It is unclear what causes the microspore to form in the film. However, they might have been produced by the solvent’s delayed evaporation during the spin-coating film-production process. This microspore may impede the PCE of PSCs due to potential high current leakage, particularly for the K-0 film.

Figure 4a,a1 show the top views of SEM images of the K-0 perovskite film, while Figure 3b–f show SEM images’ top views of the samples treated with KI (K-2 to K-10) perovskite films. All the prepared perovskite films have tightly packed multigrain morphological characteristics. Although the K-0 and KI-incorporated films have equal grain sizes, the K-2 sample has substantially larger well-packed grain particles, including many irregular tiny grains, comparatively small grain diameters, and more homogeneous particles. This indicates that some extra iodine may have evaporated during the formation of the perovskite film. This grain structure would probably make moving and removing photogenerated charge carriers from the device easier. However, as shown in Figure 3a,a1, the K-0 ternary-cation device has larger grains formed than the device with KI incorporation added to the precursor solution. As a result, grain boundaries corresponding to the device substrate can obstruct vertical-charge transport while also increasing bulk trap density via extra grain-boundary recombination centers [34,43,44]. Previous research has demonstrated that adding KI to a perovskite precursor somewhat enhances film crystallinity and grain size [13,39,45]. However, the SEM images obtained in the present study slightly refine that view in the sense that the grain sizes in the perovskite films with no KI turned out to be larger than for all perovskite films with added KI, regardless of the concentration. However, KI did progressively increase the grain size for the K-2 to K-10 absorber films, which can be observed from the SEM micrographs at a 1 μm magnification, which produced a better resolution than the 4 magnification.

Figure 3b to 3f show that KI incorporation drastically changes the surface morphology with a larger grain size in the perovskite film. Consequently, after KI incorporation, the grain boundaries were greatly reduced, effectively suppressing the influence of ion distribution among the functional layers of the device. The big grains observed in the cross-sectional SEM images in Figure 3g,h parallel the substrate, indicating that only the vertical grain boundaries are created in these images. As a result, the vertical grain boundaries in the case of the KI-incorporation devices decreased, and no additional lateral grain boundaries were introduced, as shown in Figure 4h of the cross-sectional view of the K-2 device with K^+^-incorporated perovskite films. This will effectively reduce the charge-trapping interfaces and photocurrent conduction routes in the KI-incorporated devices [34]. The SEM examination of all the prepared perovskite devices showed, as illustrated in Figure 3g,h, that the perovskite layer has a thickness of about 365.2 nm in the K-0 samples and 540.9 nm in the K-2 samples, allowing for adequate light absorption. Thus, KI addition enhances perovskite film deposition on top of the FTO/c-TiO₂/M-TiO₂ layers during device fabrication.

Additionally, it can be seen in the series of SEM images for K-2 to K-10 perovskite films that tiny particles develop at the grain boundaries after KI incorporation. The identity of these particles as BiI₃ has been established. Therefore, according to the findings, the presence of BiI₃ might reduce charge-carrier recombination, increase the open-circuit voltage (Voc) of PSCs, and provide high PCE [46,47]. A deduction is obvious from the observed reduction in the fraction of bright BiI₃ crystallites in Figure 4a. It is interesting to note that the amount of unreacted surplus BiI₃ reduces with the addition of KI. In addition, BiI₃ solubility in water is reduced due to the shorter Bi-I bond length and advanced binding strength. The surface of the perovskite layer containing BiI₃ is more resistant to humidity deterioration than other perovskite materials [2]. Consequently, upon KI incorporation, the moisture deterioration of the PSCs will be inhibited.

**Figure 5:**
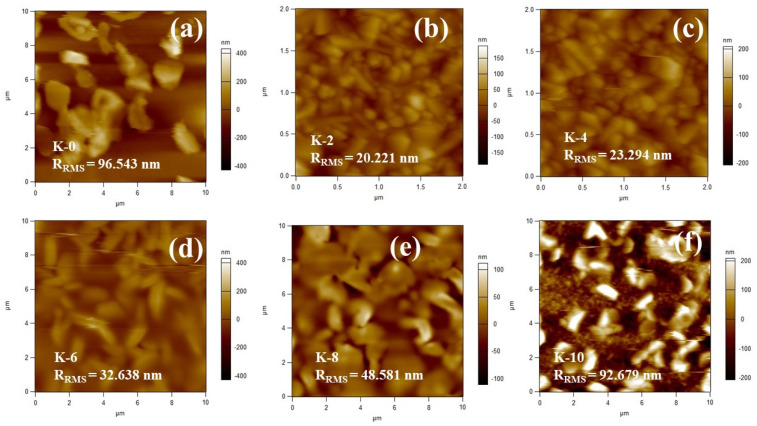
(**a**) AFM 2D topography images for deposited Cs₃Bi₂I₉ (K-0) perovskite film. (**b**–**f**) AFM 2D topography images for Cs₃Bi₂I₉ perovskite films with the incorporation of K^+^ (K-2 to K-10).

Figure 5 illustrates the use of AFM to evaluate the perovskite film’s surface topography for light trapping and surface roughness. Figure 5a–f show AFM 2D images of the hexagonal lattice perovskite morphology of the Cs₃Bi₂I₉ film (K-0) and Cs₃Bi₂I₉ film with K⁺ incorporation (K-2 to K-10). It is possible to observe a considerable increase in perovskite grain size after K^+^ incorporation, which is matched well with the SEM morphological structure. The surfaces of the Cs₃Bi₂I₉ (K-0) device and K⁺ incorporation Cs₃Bi₂I₉ devices (K-2 to K-10) differed in terms of their arithmetic root-mean-square roughnesses (R_RMS_). As shown in Figure 5a, the K-0 sample without being treated with KI has the highest R_RMS_ of 96.543 nm.

In contrast, perovskite films K-2, K-4, K-6, and K-8 have relatively lower RMS values of 20.221, 23.294, 32.638, and 48.581 nm, respectively, shown in Figure 5b–e. This suggests that the perovskite surface is altered and made smoother by KI incorporation, with 2 vol% yielding the smoothest surface, while increasing KI tends to progressively increase the surface roughness. The performance of the related devices may improve as a result of this. The untreated sample without K^+^ has small grains and ambiguous grain boundaries, as seen in Figure 5a. On the other hand, a considerable increase in grain size could be seen in the KI-incorporated samples, as shown in Figure 5b–f, making the grain boundaries apparent. It has been established that iodine is primarily responsible for the defects at the grain boundaries in perovskites. This will consequently eliminate the charge trapping caused by the defect states at the grain boundaries [13,22].

Generally, the surfaced morphology of Glass/FTO/c-TiO₂/M-TiO₂ substrates contributed to the upper roughness for all Cs₃Bi₂I₉ perovskite films. As observed in Figure 5c–f, the amount of KI is directly proportional to surface roughness as the vol.% of KI increases from 4 vol.% to 10 vol.%. This shows that a certain amount of KI is needed to enhance the overall morphological structure to lead to a better performance of Cs₃Bi₂I₉ PSCs, which agrees with the grain boundaries observed with SEM in Figure 4.

Similarly, enhancement favored higher charge-separation performances in a mesoscopically structured bismuth-based PSC-interface contact area between the layered perovskite film and the neighboring charge-carrier layer [48].

### 3.3. Perovskite-Film Optical Properties

**Figure 6:**
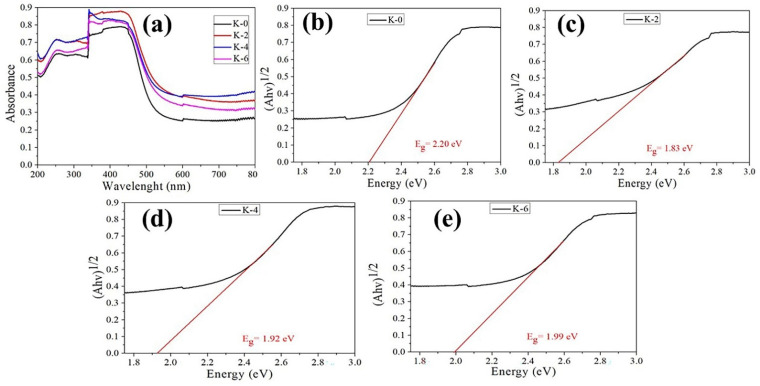
(**a**) Cs₃Bi₂I₉ perovskite film-absorption spectrum K-0 to K-10. (**b**–**e**) Tauc plots of the calculated optical-absorbance bandgaps of perovskite films K-0, K-2, K-4, and K-6.

One key factor affecting the PCE is the electron-hole recombination rate, which is inversely proportional to the defect density. Several characterizations were performed to disclose the charge recombination kinetics, which is thought to be the fundamental reason for the efficiency gain. It is common for the bandgap to shift in response to variations in the lattice constant. UV-VIS spectroscopy and PL spectroscopy were used to study the optical characteristics of all the fabricated Cs₃Bi₂I₉ films, as shown in Figure 6.

Figure 6a compares the optical absorption spectra collected over a 200–800 nm wavelength range from as-prepared Cs₃Bi₂I₉ perovskite films K-0 to K-10. All samples exhibited a clear broadband absorbance reduction range at 550–800 nm in the visible area. While K-2 had the highest visible absorbance, K-0 exhibited the least absorbance. All prepared perovskite films had an absorption edge in the visible spectrum at 450.0 nm, and their predicted optical bandgap value was 2.8 eV, which is consistent with that of Cs₃Bi₂I₉ single crystals [49]. Bandgap estimates for Cs₃Bi₂I₉ perovskite have been reported in the literature at various values, some based on an experiment. The optical bandgap of Cs₃Bi₂I₉ perovskite thin films was estimated to be 1.9 eV by Lehner et al. [50] and 2.2 eV by Park et al. [25]. Recently, Ghosh et al. used the UV-visible absorption spectra to infer an optical bandgap of 2.1 eV for Cs₃Bi₂I₉ perovskite films [51]. Inherent excitonic absorption due to the transition of the Bi^3+^ cation is responsible for the significant absorption observed in all of the generated PSCs films at a specific peak [52]. In Cs₃Bi₂I₉, when the bigger I^-^ ion is exchanged for a smaller one, the energy bandgap shrinks, causing a red shift in all-optical spectra. This suggests that the bandgap of the bismuth halide materials that took inspiration from perovskites is affected by the KI cation [53].

Tauc plots of the absorption spectra were used to evaluate the optical bandgaps (Eg), as illustrated in Figure 6b–e. The computed optical bandgaps are 2.20 eV, 1.83 eV, 1.92 eV, and 1.99 eV for the Cs₃Bi₂I₉ devices K-0, K-2, K-4, and K-6, respectively, and are in good agreement with the published theoretical and experimental data. The transition from the conduction to valence bands involves the anti-bonding Bi/6p states to the halogen (iodide) 3p/4p levels. A bandgap is produced as a result of this interaction [54].

**Figure 7:**
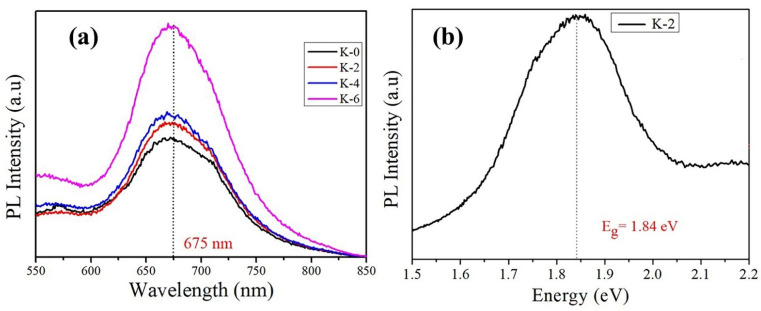
(**a**) Cs₃Bi₂I₉ perovskite device PL steady-state measurement spectra K-0, K-2, K-4, and K-6. (**b**) The PL spectra energy curve for the campion device K-2.

The optical bandgap of the as-prepared perovskite film was thus consistent with values reported earlier in this work and was further supported by the PL spectroscopy analysis. Figure 7a shows the steady-state PL spectra of Cs₃Bi₂I₉ (K-0) and Cs₃Bi₂I₉ films incorporating KI ions, which also confirmed bandgap reduction at room temperature. A characteristic peak centered on excitation at a wavelength of 669 nm (devices K-0, K-2, K-4, and K-6), corresponding to 1.84 eV, was observed for the coupling behavior of photogenerated electron-hole. The optical bandgap measured agreed with the bandgap observed using UV-visible spectroscopy (Figure 6b–e), which is attributed to band-edge excitonic irradiative illumination. As KI is introduced, the PL intensity is greatly increased when compared to the Cs_3_Bi_2_I_9_ film (K-0), which is a sign of lower defect trap states due to the higher film quality and is consistent with the blue shift of the described PL peak caused by fewer defects [55,56]. In addition, it was also found that the emission peak of the K-10 sample was much stronger than other KI-incorporation and K-0 samples. Phonon scattering and surface recombination are responsible for the overall PL-emission peaks [57].

Provided that the bandgap’s value closely matches the location of the observed maximum, the relatively large redshift of the PL signal’s maximum relative to the absorption initiation can be attributed to the achievements of this bandgap, which is located at about 1.84 eV in the best-performed device K-2, as shown in Figure 7b.

The effects of different exchange correlations on the morphological, mechanical, optical, and electrical properties of the lead-free bismuth-based ternary perovskite Cs₃Bi₂I₉ device were thoroughly investigated. However, the PL results show that Cs_3_Bi_2_I_9_ devices with KI can be included in functional solar devices.

### 3.4. Electrochemical Impedance Spectroscopy (EIS) Characterization

**Figure 8:**
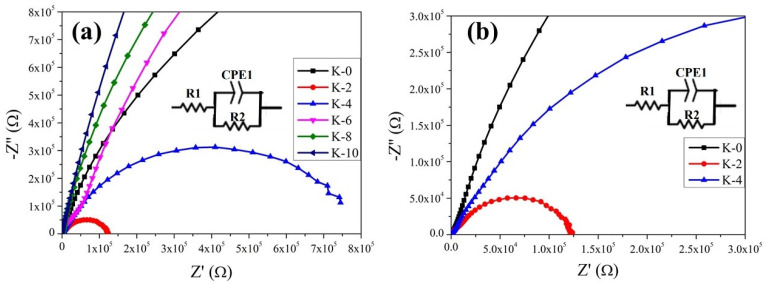
(**a**) Nyquist curves of Cs₃Bi₂I₉ devices K-0, K-2, K-4, K-6, K-8, and K-10 (inset: indicates the Nyquist curves’ corresponding circuit). (**b**) Magnification (**a**) and comparison of K-0, K-2, and K-4.

The electrochemical impedance spectrum was used to further confirm the observations on photovoltaic properties and investigate the device’s interfacial charge-transport recombination carriers’ behavior for as-prepared Cs₃Bi₂I₉ devices K-0, K-2, K-4, K-6, K-8, and K-10 under dark conditions, as shown in the Nyquist (Z’-Z”) plots in Figure 8a. Figure 8b shows a magnified comparison of K-0, K-2, and K-4 in Figure 8a. Measurements and comparisons were made between the Nyquist plots of devices with Cs₃Bi₂I₉ and Cs₃Bi₂I₉ KI incorporations, as well as the predicted curves based on a popular equivalent circuit model (inset). It shows an analogous circuit diagram, which includes series resistors (R1), compound-charge transport resistors (R2), and constant-phase elements (CPE1). Nyquist plots show that the low-frequency main arc signal reflects R2 and CPE. When considering the entire series of resistances of the gadgets, R2 was crucial. Consequently, extremes of value (both low and high) are preferred. As can be observed in Figure 8, the K-2 device has a smaller electrochemical impedance semicircle arc radius in the high to medium frequencies resulting in higher charge mobility. After the light is off, the semicircle’s diameter decreases and returns to dark conditions. According to the fitting results, the R2 of the K-2 device was more significant than that of the K-0 device. An increase in open-circuit voltage can be attributed to a decrease in the recombination rate following KI incorporation. Diffusion components may be detected, and the interfacial capacitance and charge-transfer resistance can be freely separated using the electrochemical impedance spectrum [58]. According to the various experimental tests conducted in this work, all Cs₃Bi₂I₉ devices with incorporated KI should have a better performance, but the electrochemical impedance for the K-0 device was better than K-6, K-8, and K10, and it showed lower resistance with a smaller semicircular arc. The diameter of the semicircle increased within the illumination conditions observed, which indicated the enhancement of the resistance. The resistance enhancement was correlated with reducing the capacitance in the low-frequency region [58,59].

### 3.5. Photovoltaic Properties and Perovskite-Film Device’s Stability

For bismuth-based PSCs, previous studies recommended employing dopant-free or inorganic hole transport materials [29,32,54]. According to Table 2, most PSCs made with inorganic hole transport materials, such as CuI, have a PCE of over 1%. Non-wetting hole transport layers have been shown to significantly reduce carrier recombination [60,61] and decrease heterogeneous nucleation and facilitate crystal formation with big grains. For organic and inorganic electronic materials, crystallinity significantly impacts their electrical characteristics and PCE.

**Figure 9:**
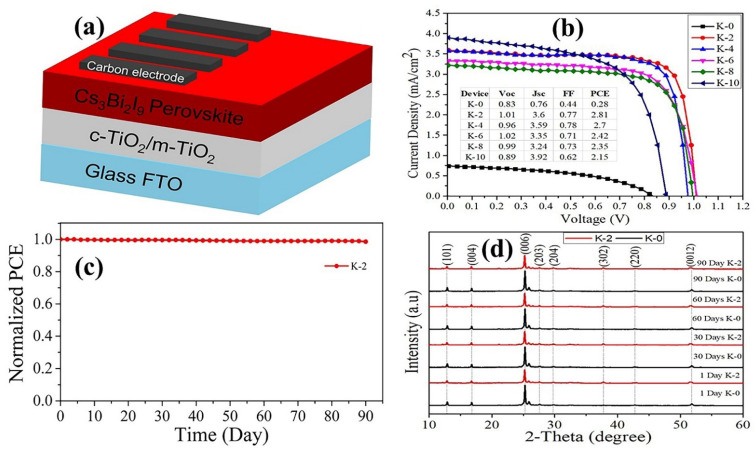
(**a**) Schematics of device architecture; (**b**) J-V curves and inserted device parameters for K-0, K-2, K-4, K-6, K-8, and K-10; (**c**) normalized PCE for K-2 device storage-time stability in ambient air from 0 to 90 days; (**d**) XRD characterization patterns from 1 to 90 days for K-0 and K-2 devices.

The photovoltaic capabilities of Cs_3_Bi_2_I_9_ and Cs_3_Bi_2_I_9_ KI incorporation as an absorber layer in a mesoscopic solar cell with architecture Glass/FTO/c-TiO₂/M-TiO₂/Cs_3_Bi_2_I_9_/carbon were examined, as shown in Figure 9a, on 120 devices.

Figure 9b presents the current-density–voltage (J-V) curves for the top-performing PSC devices with incorporated KI (K-2, K-4, and K-6) in comparison to K-0, as analyzed under the maximum-power point-tracking-simulated solar illumination of 100 mW/cm^2^ (AM 1.5G) by scanning at a rate of 100 mV/s. Initially, the photocurrent density sharply increases with the increasing KI concentration in the precursor solutions from 0.83 mA/cm^2^ for K-0 to the highest of 3.60 mA/cm^2^ for K-2, as shown in Figure 9b. The highest power-conversion efficiency (PCE) of 2.81% was obtained from the K-2 device with the open-circuit voltage (Voc) of 1.01 V, the short-circuit current density (Jsc) of 3.60 mA/cm^2^, and the fill factor (FF) of 0.77, which is lower than highest PCE reported in [17] and [26]. In contrast, the K-0 (Cs_3_Bi_2_I_9_ with KI) device performed the PCE of 0.28% with JSC = 0.76 mA/cm^2^, Voc = 0.83 V, and FF = 0.44. Compared to the K-0 device, the results for the K-2 (2 vol% KI incorporation) device significantly increase in Jsc from 0.76 to 3.60 mA/cm^2^, which is certainly attributed to a lower bandgap and higher absorbance resulting from phase transition from the P6_3_/mmc to 2-dimensional (P3m) phases with this composition architecture. The devices’ PV parameters are presented under the curves simulating their J-V behavior in Figure 9b, and it can be observed that Voc and PCE dramatically improve after incorporating KI into the perovskite layer. K-6 shows the highest Voc of 1.02 V, which is marginally better than the overall best device (K-2), whereas K-4 shows the highest FF of 78%. This implies that non-radiative trap-assisted recombination within the cell is significantly reduced, which leads to a considerable improvement in the PCE from 0.28% for K-0 to 2.81% for K-2. However, the amount of KI increases in the stoichiometric Cs_3_Bi_2_I_9_ perovskite; the Voc, Jsc (with the exception of K-10), and FF all slightly decrease with excess KI, leading to a nearly linear reduction in PCE. These values are comparable with the data from previous studies of enhanced performance in PSCs after a corresponding dose of potassium iodide [21,30,34,66]. While the relatively low PCEs of K-8 and K-10 devices were still ~ 8 times higher than for K-0, the V_OC_ values were comparable to K-0. This implies that there is an upper limit to the effective concentration of KI that can be integrated into Cs_3_Bi_2_I_9_ perovskites for the effective mobility of electron-hole recombination and undesirable grain growth of the perovskite crystals [13,27,33,34,67]. For example, the AFM surface topography images in Figure 5 show that the grain size and, subsequently, the surface roughness of K-0 and K-10 are comparable. K-10 performs better photovoltaically than K-0 because KI incorporation enhanced the morphological characterization; subsequently, the perovskite defect density is less than for K-0. On the same premise, K-2 has the smallest crystals, smoothest surface morphology, and, subsequently, the highest photovoltaic performance.

Additionally, the K-2 test device was exposed to the open air while its PCE normalized as a function of time of the device’s stability was analyzed. Figure 9c shows that the K-2 device preserved over 98% of its original PCE after 90 days, suggesting potentially improved stability. Based on these findings, it is clear that the KI-incorporated Cs_3_Bi_2_I_9_ device is superior to its counterparts [33,66,68]. To that end, if it can achieve a performance on par with a Cs_3_Bi_2_I_9_-based perovskite device, it could be a viable alternative to a Pb-based perovskite device in addressing toxicity and instability. The band orientation of Cs_3_Bi_2_I_9_-incorporated KI can be tweaked to increase carrier kinetics and film quality, leading to more a promising device performance. These data support the integration of KI into the perovskite layer as a viable technique for enhancing the long-term stability of Cs_3_Bi_2_I_9_ PSCs.

K-0 and K-2 devices were tested for stability over 90 days in ambient conditions (RH: 20%–50%, room). The K-2 devices remained reddish for the entire 90 days, whereas the K-0 device’s color began to degrade after 90 days. The XRD characteristics of these two devices are shown in Figure 9d. As depicted in Figure 9d, after 90 days of exposure under ambient conditions, the XRD patterns do not exhibit any crystalline phase transition. The samples have the same diffraction peaks as shown in Figure 3a, with a prominent intensity peak at 2θ = 12.838°, 16.713°, 25.208°, and 51.751°, which correspond to the (101), (004), (006), and (0012) planes of the Cs_3_Bi_2_I_9_ lattice and are distinctive of this perovskite phase. A slightly insignificant change in the peaks, especially at the low angles for the K-0 device, can be observed in Figure 9d. The XRD results strongly imply that adding KI to Cs_3_Bi_2_I_9_ perovskites can dramatically enhance the PSC’s stability in the ambient environment and prevent perovskite devices from decomposing under humid conditions.

## 4. Conclusions

Potassium iodide-modified, lead-free Cs_3_Bi_2_I_9_ PSC light absorbers with carbon counter electrodes were successfully synthesized via spin coating and high-temperature annealing. High-quality Cs₃Bi₂I₉ films of ultra-smooth morphology, microsized grains, and high crystallinity were realized by KI incorporation into the perovskite. The perovskites had a hexagonal crystalline phase and light absorption in the visible region.

In comparison to Cs_3_Bi_2_I_9_ PSC devices free of KI, the KI-modified Cs_3_Bi_2_I_9_ PSC devices demonstrated a considerable improvement in photovoltaic performance. The most outstanding PCE of 2.81%, with the highest Voc of 1.01 V, Jsc of 3.60 mA.cm^−2^, and FF of 77% in the study, for these-based PSCs, was attained explicitly in the solar cell containing 2 vol% of KI (K-2 device). PSCs based on Cs_3_Bi_2_I_9_ with KI incorporated into the perovskite precursor solution at a low cost and possessing a relatively high efficiency, prepared through green synthesis, demonstrating excellent stability in ambient air, and able to maintain 98% of the initial PCE after 90 days could represent the next generation. This is mainly attributed to the enhanced surface morphology, reduction in the interface energy barrier, and increased diffusion potential due to the KI. This study laid the groundwork for future advancements in bismuth-based PSC performance through a finer regulation of surface morphology/crystallinity and optimization of the interfacial carrier transport layer for efficient carrier kinetics. Therefore, the efficiency and long-term stability of the PSCs were greatly enhanced by the introduction of KI-modified internal flaws and the perovskite morphological interface of the devices. Finally, bismuth-based perovskites showed promise for the advancement in solar cell technology.

## 5. Future Prospects

Generally, the 2.2 eV wide bandgap of the Bi-based perovskite structures restricted their absorption properties. Another issue that contributed to the poor performance in comparison to other perovskite materials was the Bi-based perovskite structures’ poor surface morphology. Therefore, the following tactics could be crucial to enhancing the efficiency of Bi-based PSCs:

1. The introduction of novel device architectures may enhance the performance of PSCs.
2. Bi-based PSCs may perform better if a new charge extraction/electron transport layer is developed.
3. Bi-based perovskite structures can be made into high-quality thin films using several novel techniques, substantially enhancing their photovoltaic properties.
4. The performance of PSCs may potentially be enhanced through solvent engineering and the doping of Bi-based perovskite structures.

## Figures and Tables

**Table 1:** Lattice structures and calculated bandgaps of Cs₃Bi₂I₉ for the P6₃/mmc, C2/c, and Fm-3m structures.

Materials Id	Formula	Space Group	Crystal System	Formation Energy (eV)	E above Hull (eV)	Bandgap (eV)	Volume	Sites	Density (g/cc)
mp-624214	Cs_3_Bi_2_I_9_	*P*6_3_/mmc	Hexagonal	−0.97	0.001	2.345	1418.960	28	4.585
mp-669458	Cs_3_Bi_2_I_9_	C2/c	Monoclinic	−0.971	0	2.363	1427.997	28	4.556
mp-1113055	Cs_2_KBiI_6_	Fm-3m	Cubic	−1.092	0.041	2.682	541.588	10	3.910

**Table 2:** Published photovoltaic performances of lead-free bismuth-based (Cs₃Bi₂I₉) PSCs fabricated to date.

Cs₃Bi₂I₉ PSCs Device Structure	Voc (V)	Jsc (mAcm^−2^)	FF [%]	PCE (%)	Ref.
FTO/c-TiO₂/M-TiO₂/Cs₃Bi₂I₉/C	1.01	3.60	77	2.81	This work
FTO/c-TiO₂/M-TiO₂/photoactive film/PDBD-T/Au	0.60	7.65	78	3.59	[17]
AZO/c-TiO₂/Cs₃Bi₂I₉/CuSCN/graphite	0.37	1.43	32	0.17	[24]
FTO/c-TiO_2_/m-TiO₂/Cs₃Bi₂I₉/SpiroOMeTAD/Ag	0.85	2.15	60	1.09	[25]
FTO/c-TiO₂/Cs₃Bi₂I₉/Spiro-OMeTAD/Au	0.79	4.45	50	1.77	[26]
FTO/c-TiO₂/Cs₃Bi₂I₉/PTAA/Au	0.83	4.82	57	2.3	[26]
FTO/c-TiO₂/Cs₃Bi₂I₉/CuI/Au	0.86	5.78	64	3.2	[26]
FTO/TiO₂/mp-TiO₂/Cs₃Bi₂I₉/spiro-OMeTAD/Au	0.64	0.67	49	0.21	[38]
FTO/c-TiO₂/m-TiO₂/Cs₃Bi₂I₉/P3HT/Ag	0.26	0.18	37	0.02	[62]
FTO/c-TiO₂/m-TiO₂/m-ZrO₂/Cs₃Bi₂I₉/C	0.46	4.75	69	1.51	[63]
ITO/NiOx/Cs₃Bi₂I₉/PCBM/C60/BCB/Ag	0.75	0.51	59	0.23	[64]
ITO/PTAA/Cs₃Bi₂I₉/PCBM/AZO/Ag	0.47	1.76	45	0.37	[65]
ITO/PEDOT:PSS/Cs₃Bi₂I₉/PCBM/AZO/Ag	0.38	0.54	35	0.073	[65]
ITO/NiOx/Cs₃Bi₂I₉/PCBM/AZO/Ag	0.74	3.42	51	1.26	[65]

## Data Availability

The data presented in this study are available on request from the corresponding author.
